# Genome Analysis of Food Grade Lactic Acid-Producing Bacteria: From Basics to Applications

**DOI:** 10.2174/138920208784340731

**Published:** 2008-05

**Authors:** B Mayo, D van Sinderen, M Ventura

**Affiliations:** 1 Departamento de Microbiología y Bioquímica, Instituto de Productos Lácteos de Asturias (CSIC), 33300-Villaviciosa, Asturias, Spain; 2 Alimentary Pharmabiotic Centre and Department of Microbiology, University College Cork, Cork, Ireland; 3 Department of Genetics, Anthropology and Evolution, University of Parma, Italy

**Keywords:** Genomics, lactic acid bacteria, lactococci, lactobacilli, bifidobacteria, Streptococcus thermophilus, fermented dairy products, probiotics.

## Abstract

Whole-genome sequencing has revolutionized and accelerated scientific research that aims to study the genetics, biochemistry and molecular biology of bacteria. Lactic acid-producing bacteria, which include lactic acid bacteria (LAB) and bifidobacteria, are typically Gram-positive, catalase-negative organisms, which occupy a wide range of natural plant- and animal-associated environments. LAB species are frequently involved in the transformation of perishable raw materials into more stable, pleasant, palatable and safe fermented food products. LAB and bifidobacteria are also found among the resident microbiota of the gastrointestinal and/or genitourinary tracts of vertebrates, where they are believed to exert health-promoting effects. At present, the genomes of more than 20 LAB and bifidobacterial species have been completely sequenced. Their genome content reflects its specific metabolism, physiology, biosynthetic capabilities, and adaptability to varying conditions and environments. The typical LAB/bifidobacterial genome is relatively small (from 1.7 to 3.3 Mb) and thus harbors a limited assortment of genes (from around 1,600 to over 3,000). These small genomes code for a broad array of transporters for efficient carbon and nitrogen assimilation from the nutritionally-rich niches they usually inhabit, and specify a rather limited range of biosynthetic and degrading capabilities. The variation in the number of genes suggests that the genome evolution of each of these bacterial groups involved the processes of extensive gene loss from their particular ancestor, diversification of certain common biological activities through gene duplication, and acquisition of key functions *via *horizontal gene transfer. The availability of genome sequences is expected to revolutionize the exploitation of the metabolic potential of LAB and bifidobacteria, improving their use in bioprocessing and their utilization in biotechnological and health-related applications.

## INTRODUCTION

Lactic acid bacteria (LAB) encompass a heterogeneous group of microorganisms, which have as a common metabolic property the production of lactic acid from the fermentation of carbohydrates [1]. LAB are Gram positive, non-sporulating and acid tolerant, and belong to the *Firmicutes* (e.g., *Lactobacillus*, *Lactococcus*, *Streptococcus*, *Pediococcus*, *Oenococcus*, *Enterococcus*, *Leuconostoc*). Members of the genus *Bifidobacterium* are also Gram-positive, non-sporulating bacteria which produce lactic acid as one of their major fermentation end-products. However, taxonomically speaking they are members of the *Actinobacteria*, but will be discussed as part of this review as they are frequently used in association with LAB species in many fermented dairy products. From a biochemical perspective LAB include both homofermenters, which mainly produce lactic acid, and heterofermenters, which, apart from lactic acid, yield a variety of fermentation products such as acetic acid, ethanol, carbon dioxide and formic acid [2]. The ecological distribution of LAB is vast; they are found in a large variety of environments, including milk and dairy products, vegetable and plants, cereals and meat. Many LAB species are used for the manufacture and preservation of fermented feed and foods from raw agricultural materials in which they are either present as contaminants or deliberately added as starters in order to execute the fermentation processes. Development of these bacteria contributes to the final organoleptic, rheological and nutritional properties of fermented products [3]. Various LAB and bifidobacterial species are also commonly found among the resident microbiota of the gastrointestinal tract (GIT) and genitourinary tract (GUT) of human and animals [4, 5]. In these environments such commensals are considered to be important components of the microbiota, playing a large variety of health-promoting functions such as immunomodulation, intestinal integrity and pathogen resistance [6, 7]. For this reason, strains of some species (the majority belonging to the *Bifidobacterium* and *Lactobacillus *genera) have traditionally been used as probiotics and added as functional components to various food products [6]. Thus, the commercial exploitation of LAB and bifidobacterial species as starter and as probiotic cultures is economically very significant. Consequently, in the last 25 years research on genetics, physiology and applications of these lactic acid-producing bacteria has enjoyed explosive growth [8, 9].

One particular area that has undergone very rapid progress in the last decade is the characterization of LAB and bifidobactreial genomes. The availability of these genome sequences has improved our understanding of the fermentation pathways and biochemical routes involved in industrial and probiotic applications. This knowledge will ultimately allow a full exploitation of their fermentative capabilities, facilitating, at the same time, the genetic manipulation of these bacteria, which would lead to the use of LAB/bifidobacterial species as a cell factory for new biotechnological applications, such as expression of heterologous proteins, synthesis of food-grade additives and nutraceuticals, or as vaccine delivery systems [10, 11, 12, 13].

## GENERAL FEATURES OF LAB GENOMES

At present, more than 20 complete genome sequences of LAB and bifidobacterial strains belonging to 15 different species are available (Table **1**; http://www.ncbi.nlm.nih.gov/genomes/MICROBES/microbial_taxtree.html), while some others are at various stages of completion. LAB and bifidobacteria have relatively small genomes (average genome size of 2 Mb, with a coding capacity of 2,000 genes), with the number of genes found in a given LAB/bifidobacterial genome ranging from 1,600 to 3,000 [14, 15]. With punctual exceptions, all genomes display architectural features of a typical bacterial chromosome; i.e., co-orientation between gene transcription and DNA replication and an asymmetric bias in nucleotide composition of leading and lagging DNA strands [16].

Only a few genetic traits appear to be universally conserved among the different LAB genomes, including enzymes involved in glycolysis [14, 16]. Different genetic events (i.e., mutation, gene duplication, horizontal gene transfer (HGT), gene decay, gene loss and genome rearrangements) have been considered to contribute to the present genome shape and structure of LAB species. In fact, adaptation to nutritionally rich environments (e.g., milk, plant material, human and animal GIT) has promoted progressive gene decay but also acquisition of key genes through HGT [17, 18]. Evidence for genome decay has been observed for most sequenced LAB species, particularly in genes involved in carbohydrate metabolism, uptake and utilization. Notably, genome simplification and decay of anabolic and catabolic routes was particularly obvious following genome analysis of the yoghurt-associated, cooperative bacteria *Streptococcus thermophilus* [18] and *Lactobacillus delbrueckii* subsp. *bulgaricus* (*Lactobacillus bulgaricus*) [19], as well as in the cheese starter culture *Lactobacillus helveticus* [20]. In all these bacteria, around 10-12% of the coding genes appeared to be present as pseudogenes; i.e. non-functional genes, due to frame shifts, non-sense mutations, deletions or truncations.

In the following sections, a short description of the principal features of the genome sequences of selected LAB and bifidobacterial species used as starters and adjunct cultures in food fermentations and/or as probiotics in functional ingredients is presented. Some basic and applied aspects gathered as a result of the analysis of these genomes, in particular those of industrial and biotechnological interest will also be discussed.

### Bifidobacteria

Bifidobacteria are Gram-positive prokaryotes that naturally colonize the human and animal GIT. Although not numerically dominant in this complex ecosystem, they are considered key commensals in promoting health and well-being [6]. Among the approximately 30 recognized species of bifidobacteria [21], only the genome sequence of five strains, representing three species, are currently available: *Bifidobacterium adolescentis* ATCC 15703, *B. adolescentis* L2-32, *Bifidobacterium dentium *ATCC 27687, *Bifidobacterium longum* NCC 2705, and *B. longum* DJO10A. Of these, only the analysis of *B. longum* NCC 2705 has been published [22], accompanied with some cross references to the sequence of *B. longum* DJO10A [22, 23]. The genomes of additional bifidobacterial species/strains [for example *Bifidobacterium breve* UCC2003 (S. Leahy, M. O’Connell-Motherway, J.A. Moreno-Muñoz, G.F. Fitzgerald, D.G. Higgins, and D. van Sinderen, unpublished data), *Bifidobacterium dentium* Bd1 (M. Ventura, C. Canchaya and D. van Sinderen, unpublished results) are at various degrees of completion and detailed sequence information for some of these genomes is expected to become publicly available in the near future.

#### Bifidobacterium longum Biotype Longum NCC 2705

The 2.26-Mb genome of NCC 2705 strain is organized as a 60%-GC circular chromosome, containing 4 *rrn* operons, 57 tRNAs, 16 intact insertion sequence (IS) elements, integrated plasmid sequences [22], and a prophage-like element [24]. The genome is predicted to encode 1,727 proteins. *B. longum* has no aerobic or anaerobic respiratory components, consistent with its existence as a strict fermentative anaerobe. Several physiological traits that may explain the successful adaptation of this bacterium to the environment of the colon have been found. In particular, an unexpectedly large amount of coding capacity (>8% of the genome) appears to be dedicated to the transport (mainly ABC-type transporters) and catabolism (glycosyl hydrolases) of (mono-, oligo-, and poly-) saccharides, features shared with other colonic inhabitants, such as *Enterococcus faecium* and *Bacteroides fragilis *[25]. Many of these genes are present in seemingly self-regulated modules that appear to have arisen from gene duplication or horizontal acquisition, suggesting that *B. longum* is under strong selective pressure to acquire catabolic diversity in order to successfully compete for nutritients in the GIT ecosystem. Complete pathways for most amino acids, all nucleotides, and some key vitamins were identified; however, routes for aspartic acid and cysteine were atypical. More importantly, genome analysis provided insights into the reciprocal interactions of bifidobacteria with their hosts. Polypeptides showing homology to proteins needed for the production of glycoprotein-binding fimbriae, which are structures that may be important for adhesion and persistence in the GIT, have been identified. Furthermore, a eukaryotic-type serine protease inhibitor (serpin) that may be involved in the reported immunomodulatory activity of bifidobacteria has been identified and characterized [26].

#### Bifidobacterium breve UCC2003

The *B. breve* UCC2003 genome is the largest bifidobacterial genome so far known to be sequenced (2,422,668 bps) [6]. The circular chromosome of *B. breve* UCC2003 contains 1,828 predicted genes and 2 *rrn* operons, 54 tRNAs, 26 intact insertion sequence (IS) elements, (S. Leahy, M. O’Connell-Motherway, J.A. Moreno-Muñoz, G.F. Fitzgerald, D.G. Higgins, and D. van Sinderen, unpublished data) and a prophage-like element [24]. Similar to *B. longum* NCC2705, a significant proportion of the genome of *B. breve* UCC2003 encodes enzymes involved in carbohydrate metabolism including 40 glycosyl hydrolases whose assumed substrates represent a wide range of oligo- and poly-saccharides. Several of these glycosyl hydrolases are assumed to exert their activity outside the cytoplasm, such as an amylopullulanase, which allows growth of UCC2003 on starch and related sugar polymers [27]. The *B. breve* UCC2003 genome also contains a *fos* operon, which encodes a putative permease, a conserved hypothetical protein, and a β-fructofuranosidase. Transcriptional analysis of this operon in *B. breve* grown in the presence of different carbohydrate sources revealed its involvement in the breakdown of short-chain fructo-oligosaccharides (FOS) [27]. Furthermore, analysis of the *B. breve* UCC2003 genome showed that this organism encodes a relatively small number of phosphoenolpyruvate phosphotransferase systems (PEP-PTS), which are typically involved in for the internalization and metabolism of monosaccharides [28].

### Lactococci

Lactococci are nonpathogenic AT-rich Gram-positive LAB commonly dominant in natural niches such as spontaneous milk fermentations, on cattle, and on plant material. *Lactococcus lactis* strains, particularly representatives of the *lactis* and *cremoris* subspecies, are the main components of starters used in the economically important fermentation of milk into cheese [29], contributing to acidification, prevention of undesirable bacterial growth, and flavor formation through their proteolytic and amino acid conversion pathways [30]. *L. lactis* is probably the best characterized LAB species and, for this reason, has become a paradigm for fundamental physiological and genetic research. Lactococci have recently been targeted for novel biotechnological applications, such as expression of heterologous proteins, synthesis of food-grade additives and nutraceuticals, and in vaccine delivery [31, 32]. At present, the sequences of three *L. lactis* strains are publicly available: the plasmid-cured strains *L. lactis* subsp. *lactis* IL1403 [33] and *L. lactis* subsp. *cremoris* MG1363 [34], and the plasmid-containing starter strain *L. lactis* subsp. *cremoris *SK11 [15].

#### Lactococcus lactis IL1403, MG1363 and SK11 Genomes

The genomes of the *L. lactis* strains range from 2.3 to 2.6-Mb in size, encoding 2,300 to 2,500 proteins, 6 *rrn* operons and 62 tRNA genes. A variable number of prophages (4 to 6) were encountered in these strains, as well as an extremely rich panoply of IS elements (from 43 to 130). These elements do not appear to be distributed at random. In fact, the non-random distribution of IS in *L. lactis* IL1403 suggests that its chromosome is the product of a recent recombination event between two closely related genomes [33, 35]. In contrast, the concentration of one-fifth of the 71 IS elements in a specific 56-kb region in the *L. lactis* MG1363 strain was interpreted as an integration hotspot region [34]. This integration hotspot carries genes that are typically associated with lactococcal plasmids and a repeat sequence specifically found on *L. lactis* plasmids and in the so-called “lateral gene transfer hotspot” of the *S. thermophilus *genome [18]. The chromosomes of MG1363, IL1403 and SK11 show extensive gene synteny, if a large chromosomal inversion previously described in strain MG1363 [36] is taken into account. The parent strain of MG1363 was subjected to a prophage curing strategy [37], but it still carries four remnant/satellite phages and two apparently complete prophages [34]. A complete set of late competence genes was found in all sequenced strains, including a gene equivalent to *comX* of *Streptococcus pneumoniae*, which encodes the ECF-type σ-factor necessary for transcription of competence genes. This suggests that this species may possess the ability to develop natural competence for transformation, provided that appropriate physiological conditions are met. Surprisingly, the functions necessary for aerobic respiration were also found to be encoded on the genome. Thus, *L. lactis* may carry out oxidative phosphorylation if exogenous protoporphyrinogen is provided. This capability, which has been analyzed in detail [38, 39], is the basis for a completely new industrial process of producing lactic starters [40].

Interest in non-dairy *L. lactis* is increasing due to the search for unique flavor-forming capabilities and production of novel broad-range antimicrobials [41]. Recently, the low-coverage genome sequencing of two *L. lactis* strains isolated from plants has allowed a comparison of their genomes to those of dairy strains [42]. Adaptation to grow on plant substrates was evident from the presence of gene sets for the uptake and degradation of complex plant polymers such as xylan, arabinan, glucans, fructans, but also for the uptake and conversion of typical plant cell wall components such as α-galactosides, β-glucosides, arabinose, xylose, galacturonate, glucuronate and gluconate [42].

### Lactobacilli

Lactobacilli are a broad, morphologically-defined group of LAB characterized by the formation of lactic acid as a sole or main end product of carbohydrate metabolism. The lactobacilli are Gram-positive, non-spore-forming rods or coccobacilli with a GC content usually below 50 mol %. More than one hundred species of lactobacilli are recognized at present [43,  http://www.ncbi.nlm.nih.gov/Taxonomy]. They are strictly fermentative (either homo- or heterofermenters), aerotolerant or anaerobic, aciduric or acidophilic, and have complex nutritional requirements (carbohydrates, amino acids, peptides, fatty acid esters, salts, nucleic acid derivatives, vitamins) [43]. The nutritional requirements of lactobacilli are a reflection of their various habitats, which are typically rich in carbohydrate- and protein-containing substrates. They are found on plants, dairy and meat products, and as components of the microbiota of the animal and human GIT [44].

Lactobacilli are important starters and adjunct cultures in the production of foods that require lactic acid fermentation, notably dairy products (yogurt and cheese), fermented vegetables (olives, pickles, and sauerkraut), fermented meats (salami, sausages), and sourdough bread and other cereal-based food commodities [3]. Although less numerous than bifidobacteria, lactobacilli inhabiting the GIT of animals are thought to exert pivotal roles in the establishment and maintenance of a properly functioning GIT [7], and a number of strains have been used as probiotics for more than 70 years [45]. Beneficial effects attributed to probiotic lactobacilli include colonization of intestinal and genital mucosa [46, 47], inhibition of pathogens [48, 49], immunomodulation [50] and cholesterol assimilation [51]. It is therefore not surprising that most of the genomes from lactobacilli that have so far been sequenced are of human intestinal origin.

#### Lactobacillus acidophilus NCFM

*L. acidophilus* NCFM is a probiotic human isolate surviving GIT passage, which has been produced commercially since 1972 [52]. The complete genome is 1,993,564 bp and devoid of plasmids. The average GC content is 34.71% with 1,864 predicted ORFs, of which 72.5% have been functionally classified [17]. Four *rrn* operons were found on the NCFM genome sequence and 61 tRNA genes. Nine phage-related integrases were also predicted, but no complete prophages were found. However, three unique regions designated as potential autonomous units (PAUs) were identified. These units resemble a singular structure, bearing characteristics of both plasmids and phages. Analysis of the three PAUs revealed the presence of two R/M systems and a killer protein of a possible prophage-maintenance system. A Clustered, Regularly Interspaced Short Palindromic Repeat (CRISPR) locus containing 32 nearly perfect 29-bp repeats was discovered. Similar DNA spacers have been found in more than different 40 microorganisms including *E. coli *[53]. These have been shown to be involved in resistance to bacteriophages. Furthermore, the chromosomal locus for lactacin B, a class II bacteriocin previously isolated and characterized from this strain [54], was identified. *In silico* analysis of the genome of the NCFM strain indicated that this strain has the potential to synthesize just three amino acids (cysteine, serine and aspartate). Congruently, the genome is predicted to encode an array of ABC-transporters (nine) for amino acids and oligopeptides, separate di- and oligo-peptide transport systems, and many (i.e. 22) amino acid permeases [17]. For protein degradation and peptide utilization, the organism is predicted to encode 20 putative peptidases/proteases, including homologs of PrtP and PrtM, a proteinase system for extracellular casein degradation in *L. lactis*. Various gene clusters coding for the metabolism of a variety of carbohydrates, including FOS and raffinose, were present on the NCFM chromosome, often accompanied by transcriptional regulator-encoding genes belonging to the *lacI* family. Finally, several genes that specify mucus- and fibronectin-binding proteins, presumably implicated in adhesion to human intestinal cells, were also identified. These features are likely to contribute to the organisms’ gastric survival, promoting interactions with the intestinal mucosa.

#### Lactobacillus bulgaricus ATTC 11842

*L. bulgaricus* is a representative of the LAB species used worldwide for yogurt production in association with *S. thermophilus*. The size of the *L. bulgaricus* genome is around 1.8 Mb with an overall GC content of 49.7% [19]. An intriguing feature of the replication terminus region of the ATTC 11842 genome is the presence of a 47.5-kbp inverted repeat, representing an extremely rare structure in bacterial genomes. In contrast to many other LAB species, the *L. bulgaricus* genome does not contain any prophage. A relatively high number of *rrn* operons (9) and tRNA genes (95) were identified, suggesting that the genome has undergone a recent size reduction. This is further supported by the presence of a substantial number of pseudogenes (270 in total), various incomplete metabolic pathways, and relatively few regulatory components. A much higher GC content at codon position three than expected on the basis of the overall GC content suggested that the composition of the genome is rapidly evolving toward a higher GC content [19]. This may be the result of its adaptation to milk from a plant-associated habitat, as suggested by the presence of complete and incomplete PTS and other sugar transport systems and hydrolytic enzymes. In protocooperation with *S. thermophilus,* the loss of superfluous functions might be advantageous in the stable protein and lactose-rich milk environment [18, 19].

#### Lactobacillus helveticus DPC 4571

*L. helveticus* belongs to the *L. acidophilus-L. delbrueckii* group and is frequently used in dairy technology as a starter or adjunct culture to reduce bitterness and to increase flavor notes in cheese [29]. The complete genome sequence of the cheese culture *L. helveticus* DPC 4571 consists of 2.08 Mb with an average GC content of 37.73% [20]. It contains four *rrn* operons, 73 tRNA genes, about 1,600 protein-encoding genes and a large number of pseudogenes. Seventy five percent of the predicted ORFs in DPC 4571 have orthologues in the *L. acidophilus* NCFM genome. Not surprisingly, these two species share 98.4% of their 16S rRNA gene sequences [43]. The same relatedness was observed by constructing a phylogenetic supertree with 47 ribosomal proteins [20]. A remarkable and rather astonishing feature of the *L. helveticus* DPC 4571 genome is the presence of 213 IS elements, belonging to 21 different classes [20]. In spite of this, IS-associated gene deletion and decay was not clearly evident for the majority of genes lost. Furthermore, the extensive whole-genome conservation between *L. helveticus* and other sequenced intestinal lactobacilli suggests that the IS elements did not promote (frequent) genomic rearrangements. A genomic island of 100-kbp characterized by a GC content of 42% and flanked by IS elements and unique 12-bp direct repeats is a firm candidate of adaptative HGT in this bacterium. Although non probiotic, certain *L. helveticus* strains have been shown to exert beneficial effects by production during milk fermentation of bioactive peptides with antihypertensive and immunomodulatory properties [55]. The DPC 4571 proteolytic system was found to be represented by more than 24 genes. The products of these genes showed significant homology to known peptidases and, while clear homologs of several of these had been reported previously, novel enzymes of interest to the dairy industry (i.e. those with similarity to PepE, PepQ, PepT or PepD) were also identified.

#### Lactobacillus johnsonii NCC 533

*L. johnsonii* NCC 533 is a member of the “acidophilus” group of intestinal lactobacilli that has been extensively studied for their probiotic activities, including pathogen inhibition, epithelial cell attachment, and immunomodulation. The *L. johnsonii* NCC 533 genome is 1.99-Mb in size with a GC content of 34.6%. It contains six *rrn* operons at four loci, 79 tRNAs, 14 complete IS elements from three known families, and two complete prophages [56]. Strikingly, the organism completely lacks genes encoding biosynthetic pathways for amino acids, purine nucleotides, and most cofactors. In apparent compensation, a remarkable number of uncommon and often duplicated amino acid permeases, peptidases, and PEP-PTSs were discovered, suggesting a strong dependency of strain NCC 533 on the host or other intestinal microbes to provide simple monomeric nutrients. Thus, competition of *L. johnsonii* with bifidobacteria and bacteroides seems unlikely; instead this species appears to be better adapted to the upper part of the GIT, where amino acids, peptides, and mono- and oligosaccharides are abundant. Genome analysis also predicted an abundance (>12) of large and unusual cell-surface proteins, including fimbrial subunits similar to those of pathogens, which may be involved in adhesion to glycoproteins or other components of mucin, a characteristic expected to support persistence in the GIT. In this stable environment, *L. jonhsonii* was found to direct transcription through a single (primary) sigma factor. Three bile salt hydrolases and two bile acid transporters, which might also be critical for GIT survival, were also detected. *In silico* genome comparison with the genome sequence of the closely related *L. gasseri* revealed extensive synteny. Moreover, the DNA sequence of many housekeeping genes of these two bacteria showed a high degree of similarity.

#### Lactobacillus plantarum WCFS1

*L. plantarum *WCFS1 is a single colony isolate from *L. plantarum* NCIMB 8826, originally isolated from human saliva [57]. The genome of the WCFS1 strain is the largest LAB genome analyzed so far. It consists of a circular chromosome and three plasmids (of 1.9, 2.3 and 36.0 kbp). It is 3.3-Mb in size with an overall GC content of 44.5%, and contains 3,052 predicted protein-encoding genes, five *rrn* operons evenly distributed around the chromosome and 62 tRNA genes [57]. The *L. plantarum* genome encodes complete pathways for biosynthesis of most amino acids, except for branched-chain amino acids (valine, leucine and isoleucine). The genome also lacks an extracellular protease equivalent to PrtP of *L. lactis*. Consistent with the classification of *L. plantarum* as a facultative heterofermentative lactic acid bacterium, its genome encodes all enzymes required for the glycolysis and phosphoketolase pathways; components of these two routes appear to belong to the class of highly expressed genes in this organism (evident from their codon-adaptation index). Moreover, *L. plantarum* encodes a large pyruvate-dissipating potential, leading to various fermentation end-products. *L. plantarum* is encountered in many different environmental niches, and this flexible and adaptive behaviour is reflected by the relatively large number of regulatory, transport (including 25 complete PEP-PTS sugar transport systems), and stress-related proteins. More than 200 extracellular proteins are predicted to be encoded by the *L. plantarum* genome, of which many are assumed to be bound to the cell envelope. A large proportion of the genes encoding sugar transport and utilization, as well as genes encoding extracellular functions, are clustered in a 600-kb region near the origin of replication. Many of these genes display deviation of nucleotide composition, consistent with a foreign origin. Kleerebezem *et al*. [57] called this chromosomal segment “a lifestyle adaptation region”, suggesting that many of the proteins encoded by this region provide adaptive properties to *L. plantarum*. For the first time in a LAB strain, a gene cluster predicted to be involved in the synthesis of a non-ribosomal peptide was found, although the assumed peptide-like product(s) remain uncharacterized.

#### Lactobacillus sakei 23K

*L. sakei* is a psychrotrophic lactic acid bacterium which is naturally found on fresh meat and fish, but especially on fermented meat products where it is widely used as a starter [3]. The *L. sakei* 23K genome is 1.8-Mb in size with a GC content of 41.25 % [58]. It contains 7 *rrn* operons and 63 tRNA genes. Sequences of 12 IS elements from four families and a prophage remnant were also observed. Consistent with life in a protein-rich environment, the genome sequence of 23K revealed auxotrophy for all amino acids except aspartate and glutamic acid. Its specialized metabolic repertoire includes scavenging of purine nucleosides that may improve its competiveness on raw meat products. Many genes appear to be responsible for coping with the harsh conditions of food processing (antimicrobial substances, high salt concentration, changing redox conditions and oxygen levels). In comparison to intestinal lactobacilli, *L. sakei* and *L. plantarum* are far better equipped to cope with changing redox conditions and oxygen content, although *L. sakei* appears to deal more effectively with toxic oxygen reactive compounds [58]. Apparently, iron and heme acquisition correlates with this resistance, an ability characteristic of pathogenic bacteria. Genes potentially responsible for biofilm formation and cellular aggregation have been found in the *L. sakei* genome (including four proteins with a LPXTG motif and 15 having a WXL-like domain), which may assist the organism in colonizing meat surfaces, were also identified.

#### Lactobacillus salivarius subsp. salivarius UCC118

*L. salivarius* subsp. *salivarius* is a mucosa-associated bacterium present in the faeces, intestinal mucosa, tongue and rectum of around 10% of the human infant and adults [59]. *L. salivarius* is part of a distinct clade at the periphery of the genus *Lactobacillus* [60]. The UCC118 strain isolated from the terminal ileum is a bacteriocin-producing strain with probiotic characteristics [61]. The genome of *L. salivarius *subsp. *salivarius *UCC118 is 2.13-Mb in size, and is comprised of two large replicons [a 1.83 Mb chromosome and a 242-kb megaplasmid (pMP118)] and two smaller plasmids [of 20.4 kbp (pSF118-20), and 44 kbp (pSF118-44)] [44]. Seven *rrn* operons and 78 tRNAs, representing all 20 amino acids, were also detected on the genome. Two intact prophages and two remnant phage-related sequences and 43 copies representing 16 diferent classes of IS elements are also present. The genome sequence indicated an intermediate level of auxotrophy compared to other sequenced lactobacilli. The circular chromosome of *L. salivarius* UCC118 is the smallest *Lactobacillus* chromosome so far sequenced (57.6 kbp smaller than that of *L. sakei* and 165.5 kbp smaller than that of *L. johnsonii*). Megaplasmids of this size had not previously been characterized in LAB, but were shown to be widely distributed among different *L. salivarius* strains belonging to both *salivarius* and *salicinius *subspecies (sizes ranging from 100- to 380-kbp) [44]. More recently, even bigger plasmids have been identified in other intestinal lactobacilli [62]. No single-copy essential genes were present on the megaplasmid. However, contingency amino acid metabolic genes and carbohydrate utilization genes, including two genes for completion of the pentose phosphate pathway (a finding that groups the species among the facultative heterofermentative lactobacilli), were encoded by the megaplasmid. Furthermore, this plasmid harbors genes for production of the Abp118 bacteriocin [63], a bile salt hydrolase, a presumptive conjugation locus, and other genes potentially relevant for probiotic properties. Notably, bacteriocin production was recently demonstrated to be the primary mediator of *in vivo* *L. salivarius *UCC118 protection in mice against the pathogenic organism *Listeria monocytogenes* [64].

### Leuconostoc

The genus *Leuconostoc* comprises Gram-positive cocci that are claimed to be taxonomically and ecologically related to *Lactococcus* species. However, leuconostocs are obligate heterofermenters and produce extracellular polysaccharides in sugar-rich media [65]. Two species are of industrial significance, namely *Leuconostoc mesenteroides* subsp. *cremoris* and *Leuconostoc lactis*, which are both frequently included as starters and adjunct cultures in the production of cheeses and butter. Leuconostocs grow slowly, especially in milk, so they are not important for lactose fermentation, but they are critical for the production of flavor compounds through utilization of citrate to produce diacetyl [66].

#### Leuconostoc citreum KM20 Genome

*L. citreum* is a dextran-producing species that can be found, as many other *Leuconostoc* species*,* in fermented foods and feeds of plant and dairy origin, such as cheese, pickles, sauerkraut, and cabbage [65]. The K20 strain was originally isolated as a dominant microorganism from Kimchy, a popular fermented Korean commodity made from a variety of spicy vegetables, for which it is used as a starter [67]. *L. citreum* KM20 can suppress growth of many pathogenic microorganisms and has been found to be cytotoxic to HT-29 cells. Consequently, this strain has also been considered as a probiotic. The *L. citreum* KM20 genome consists of 1,796,248 bp with a collective 39% GC content [68]. Besides the single chromosome KM20 harbors four circular plasmids of 38.7, 31.5, 18.0, and 12.2 kbp. The entire genome showed 1,820 protein-coding genes (1,702 in the chromosome and 118 in plasmids), four rRNA operons and 67 tRNA genes. Complete phages were not found, but the genome contains five copies each of IS elements belonging to the IS*3* and IS*30* families [68]. Genome analysis revealed a complete gene set for heterolactic fermentation *via *the phosphoketolase pathway with an incomplete tricarboxylic acid cycle. A vast array of hydrolases and carbohydrate transport genes were observed, which agrees well with its association with plant-associated material. In addition, multiple genes for dextransucrases and alternansucrases and a plasmid-encoded, cell wall-anchored protein with five putative mucus-binding domains. This protein may be related to the probiotic properties of KM20 strain.

#### Oenococcus oeni

*O. oeni *is the only species of the genus *Oenococcus* [69]. It is an acidophilic member of the *Leuconostoc* branch, indigenous to grapes and other plant-related environments. Together with lactobacilli, pediococci, and *Leuconostoc* species, *O. oeni* is responsible for the malolactic fermentation in wine and cider [70]. This bacterium has rarely been associated with off-flavors and undesirable metabolites in fermented beverages. Consequently, it is frequently used as a malolactic starter [71].

#### O. oeni PSU-1 Genome

The complete genome of the natural plasmid-free *O. oeni* PSU-1 strain is approximately 1.7-Mb in size with a GC content of 38%. Around 1,700 ORFs have been predicted from the sequence, of which 75% have been functionally classified [15, 72]. The genome size is very similar to that of strain IOE 8413 (ATCC BAA-1163), whose genome sequencing is ongoing [14]. Only two *rrn* operons in opposite orientation have been found and 43 tRNA genes scattered through the chromosome (although 15 of them are clustered at one location), representing all 20 amino acids. As an obligate heterofermentative lactic acid bacterium, the genome of *O. oeni* encodes all enzymes for the phosphoketolase pathway. Moreover, genes related to flavor modification in wine, in particular those involved in the malolactic fermentation capacity (*mleAP* and *mleR*) and citrate utilization (citrate lyase gene cluster, the butanediol pathway and other genes) have been identified. Indeed, five genes homologous to the malate permease and one to the malate decarboxylase can be found on the PSU-1 genome. Thus, *O. oeni* possesses more genes in this cluster of orthologous groups of proteins (COG) than any other LAB, probably reflecting its adaptation to malate rich environments [15]. Various stress-related systems have also been identified which are thought to assist *O. oeni* to survive the harsh environment of wine and cider, including malate, citrate and amino acid conversion enzymes, class I heat shock genes (*groESL* and *dnaK* operons), a putative F_0_-F_1_ ATPase system (*atpBDFHAGDC*), and other genes involved in stress responses (*clpX, clpLP, trxA, ftsH*, and *omrA*) [72].

#### Streptococcus thermophilus

The genus *Streptococcus* comprises several harmful pathogenic species, such as *Streptococcus pyogenes* and *S. pneumoniae*, but also a single Generally Regarded As Safe (GRAS) species, *S. thermophilus. S. thermophilus* is closely related to the *S. salivarius* found in the human oral cavity, of which it was considered until recently a subspecies. *S. thermophilus* is a relatively coherent and homogenous species with a low level of nucleotide polymorphism among strains, suggesting it has recently emerged. At present, it is used extensively in the food industry for the manufacture of many dairy products (yogurt, hard cooked cheeses of the Italian and Swiss types, soft cheeses, etc.), and it is considered the second most important industrial dairy starter after *L. lactis *[29].

#### S. thermophilus CNRZ 1066 and LMG 13811 Genomes

The genome sequences of two *S. thermophilus* strains, those of CNRZ 1066 and LMG 13811, have been determined and analyzed [18]. These have been compared to genome sequence of *S. thermophilus* LMD9 [15]. The genome size of all three strains is nearly identical (around 1.8-Mb) with a GC content of 39%. Sixty-seven tRNA genes were found and six *rrn* operons. Access to these three genome sequences of *S. thermophilus* has allowed a better understanding of the evolutionary path followed by this species [73]. *S. thermophilus* and its pathogenic relatives still share a substantial part of their overall physiology and metabolism. *S. thermophilus* seems to have evolved mainly through loss-of-function events mirroring the dairy niche, resulting in the absence of most streptococcal virulence-related genes such as those involved in cell adhesion, and host invasion or escape from the immune system. However, the presence of numerous pseudogenes (around 10%) also suggests an ongoing regressive evolution. The detailed *in silico* investigation of its cellular metabolism illustrates that evolution has shaped the *S. thermophilus* genome by selection for optimal growth in milk. Notably, *S. thermophilus* has maintained a well-developed nitrogen metabolism, while its sugar catabolic abilities are strongly degenerated (two of the most highly decaying functional groups relate to carbohydrate hydrolysis, uptake, and fermentation). Additionally, *S. thermophilus* shares its ecological niche with other LAB such as *L. bulgaricus*, resulting in specific metabolic cooperation, which is either revealed by the maintenance of dedicated pathways (e.g., folate and formate production) or by the loss of key metabolic functions provided by the symbiotic partner (e.g., extracellular proteolytic activity for casein hydrolysis). Although gene decay is obvious in the *S. thermophilus* genome, numerous small genomic islands seem to have been acquired by HGT process. These regions encode a number of important adaptive traits, which are of industrial relevance such as polysaccharide biosynthesis (*eps, rgp*), bacteriocin production (*blp, lab*), restriction-modification systems or oxygen tolerance. *S. thermophilus* is considered a non-competent organism, although the *S. thermophilus* genome contains all late competence genes, a situation that is similar to that observed for *L. lactis*. In addition, it contains a *comX*-like gene coding for a typical peptide pheromone-dependent two-component system that is similar to the competence control loci of *Streptococcus mutans* and *S. pyogenes *[74]. The regulatory pathway controlling expression of key components of competence in *S. thermophilus* has recently been reported [75, see also below for a general discussion on natural transformation in LAB].

## EVOLUTIONARY ASPECTS

Selective pressure and competitiveness have been driving the evolution of LAB species in the nutrient-rich environments they inhabit. Not surprisingly, the analysis of different LAB genomes has revealed well equipped organisms for a wide range of metabolic activities, defense and stress responses, specifically needed to live, reproduce and survive in plant-derived materials, diary and meat products, and animal and human mucosa. Given the close phylogenetic relationships of these organisms, comparison of gene content across the species and reconstruction of ancestral gene sets indicate that adaptation to nutrient-rich environments has for most species promoted a combination of extensive gene loss (or degradation of dispensable genes) from ancestral bacterial types [15, 17, 18, 22, 56, 57].

Ancestral gene loss and metabolic simplification contrasts with lineage-specific duplication and/or acquisitions of unique (key) genes *via* HGT. Plasmid-encoded genes, which, for instance, account for up to 5% of the total gene content in *L. lactis* [76], are essential for growth of LAB species in specific environments. All LAB genomes contain IS elements and transposons, varying from approximately 0.2% of the genome in *L. gasseri* [15] up to nearly 10% in *L. helveticus* [20]. Sometimes, perhaps simplistically, viewed as genomic parasites, IS and transposons are thought to contribute substantially to the generation of genetic diversity, thus promoting adaptation of bacteria [77].

From an evolutionary perspective, milk in particular is a very recently developed niche, as compared to plants, and adaptation of what probably were plant-associated bacteria to the dairy environment seems to be a plausible evolutive event. The analysis of the genomes of the two *L. lactis* plant isolates and their comparison to those of dairy strains has provided a first view of the molecular basis of adaptation of this bacterium to plant and milk environments A high synteny was found between the genomes of dairy and plant isolates, but numerous genes were identified in the sequences of the plant isolates that do not have homologs in the gene complement of dairy *L. lactis* strains. By calculating the GC content of unique genes and gene clusters of the plant isolates and comparing them to their best hits, Siezen *et al*. [48] showed that most of the genes have a GC content close to or slightly lower than the 35% average for *L. lactis* genomes, while their best BLAST hits generally have a higher GC content (except for the clostridial best hits, which have a lower GC content). This strongly suggests that most of these genes and gene clusters that appear to be specifically associated with the genomes of lactococcal plant isolates were in fact not acquired by lateral gene transfer. Rather, they seemed to be more ancient and appear to have been lost from the genomes of the diary lactococci.

Certain practices in the dairy industry appear to have caused specific adaptation of some bacteria to particular milk products. This is thought to be the case of *L. delbrueckii, L. helveticus* and *S. thermophilus*, whose closest relatives are represented by commensal and pathogenic bacteria from the human and animal GIT. Strains of these species are adapted to grow in milk under the stringent conditions that are used to make dairy products such as yoghurt and cooked cheeses [78, 79]. In fact, they have never been isolated from traditional dairy products that do not undergo heating [80]. Adaptation to milk of *S. thermophilus* has resulted in genome degradation of genes that are dispensable for growth in this medium (including many involved in pathogenesis) and acquisition by horizontal gene transfer of genetic traits dedicated to efficient exploitation of milk’s nutrients [76]. As an example, a specific synporter for lactose found in *S. thermophilus* is absent from its pathogenic relatives. In common with *S. thermophilus*, adaptation of *L. helveticus* to the dairy niche involved gene decay of transport proteins, energy metabolism genes, regulators and amino acid metabolic genes, while lateral gene transfer events provided this bacterium with specific genes for fatty acid biosynthesis, restriction endonucleases and amino acid metabolic genes [20]. Gene gain has also been proposed for the acquisition by *L. bulgaricus* and *L. lactis* of a 17 kbp region of DNA, containing multiple copies of IS*1191* and a mosaic of genes devoted to generate essential nutrients from milk components [81]. A unique copy of *metC* was identified among the genes identified on this 17 kbp fragment. This gene endows LAB species with the ability to synthesize methionine, a rare amino acid in milk. Many LAB strains of different species harbor plasmids of sizes ranging from about 2-kbp to more than 300-kbp [62, 82], which are considered adaptative tools. However, apart from plasmids, most species seem to harbor chromosomal regions dedicated to the incorporation of adaptative (exogenous) DNA (the so called “life style island” or “hotspot islands”) [18, 20, 23, 35, 57].

## IDENTIFICATION OF EXISTING AND NOVEL BIOCHEMICAL ROUTES

Manipulation of fermentation pathways of lactic acid-producing bacteria to obtain a better understanding of their biochemical routes, or to improve the bacterium’s efficiency in industrial applications, was undertaken soon after the development of the first genetic engineering tools [8, 11]. As microbial genomes encode the various options determining metabolism, physiology, biosynthetic capabilities, and adaptability to varying conditions and environments of the organisms, the availability of genome sequences has significantly expanded our capabilities to exploit these microorganisms metabolic and bioprocessing potential [83] and their contribution to health and well-being [14]. The metabolic abilities of *B. longum* NCC 2705 have been reconstructed *in silico* from its genome sequence [22]. This analysis revealed that this strain possesses genes for the synthesis of at least 19 amino acids from ammonium, pyrimidine and purine nucleotides from glutamine, and most enzymes for the synthesis of folic acid, thiamin and nicotinate [22]. Genomic information can also be useful for the formulation of species- or strain-specific prebiotics to enhance growth of beneficial populations (see below). In that sense, the genome sequence of *B. breve* UCC2003 has been used to formulate a chemically-defined medium for this bacterium to undertake fundamental studies [84].

### Glucose Uptake Systems in *L. lactis*

Galactose and lactose are not always desirable in food products, because of lactose intolerance, and the involvement of galactose in cataract occurrence in certain individuals in combination with alcohol intake [85]. These considerations led to an attempt to engineer *L. lactis* into a glucose-producing cell factory. A strategy was designed to remove all glucose-PTS import activities. The glucokinase gene was also deleted to prevent glucose (entering the cell through non-PTS) from being phosphorylated and then processed *via *glucolysis. However, a glucokinase (Δ*glk*), EII^man/glc^ (Δ*ptn-ABCD*) double mutant was still capable of growth in glucose [81], which thus suggested that a third transport system is responsible for glucose uptake. DNA microarrays were used to investigate the transcriptomes of *L. lactis* wild type and mutant strains. This technique unambiguously demonstrated that EII^cel^ (*ptcBAC*), previously thought to be involved in the transport of cellobiose, was the only up-regulated operon in the double knock-out strain. A triple mutant was constructed and selected on galactose. This strain was indeed shown to be incapable of growth on glucose. Subsequently, the lactose plasmid pMG820 [37], carrying the genes for the lactose-PTS and the tagatose-6-phosphate pathway, was introduced by conjugation into the mutant. The new strain was shown to grow slowly on lactose, but it efficiently fermented the galactose moiety, while excreting glucose at equimolecular levels to the lactose internalized. No galactose was detectable in the growth medium, as opposed to the wild type strain which produced low levels of this sugar. Indeed, the strain was able of growing in skim milk where it produced up to 22 mM of glucose.

### Carbohydrate Degradation and Utilization

As obligate fermenters, LAB and bifidobacteria need to use carbohydrates as a source of energy for growth and acidification. Not surprisingly, a vast array of diverse carbohydrate-degrading (glycosyl hydrolases) enzymes have been encountered scattered throughout their genomes. However, as a result of specialization and adaptation, significant differences exists in the number and types of glycosyl hydrolases among the different LAB (and bifidobacterial) species. β-galactosidases, β-phospho-galactosidases and β-glucosidases are involved in the utilization of lactose in the milk environment [86], while α-galactosidases are responsible in plant-derived environments for the breakdown of α-galactosides [87], compounds that can not be degraded by monogastric animals, thus causing flatulence and other intestinal malfunctions. Furthermore, oligosaccharide-type galactosides are recognized as bifidogenic factors and prebiotics, meaning that LAB and bifidobacteria must present a specific degrading machinery [88]. Moreover, carbohydrate hydrolases are also involved in the intestinal tournover of the glycocalix material, which may represent colonizing and competence factors for bacteria [25]. Glycosydases from intestinal microorganisms may also participate in the deglycosylation of phytoestrogens from plant materials of the diet (isoflavones, lignans, and fenylflavonoids), which in several cases is an essential step in the formation of biologically-active compounds from precursors, such as equol, enterodiol, enterolactone and 8-phenylnaringenine [89]. Alternatively, glycosydases may also contribute to the activation of pro-carcinogenic molecules that may ultimately be harmful for the host [90]. In this respect, β-glycosydases release aglycones with mutagenic and/or carcinogenic activities and β-glucuronidases may re-activate in the intestine conjugated carcinogens that are produced by the liver [91].

As a striking example of carbohydrate-degrading activities, it is predicted that bifidobacterial genomes encode a large number (greater than 40) of glycosyl hydrolases, some of which are assumed to exert their activity outside the cytoplasm; e.g. amylopullulanase that allows growth of UCC-2003 on starch and related sugar polymers [27]. β-fructofuranosidases are among the enzymes reported to allow the digestion of a particular group of bifidogenic carbohydrates, the FOS [92]. These enzymes specifically catalyze the hydrolysis of the β-(2-1)-glycosidic bond between glucose and its neighboring fructose moiety in sucrose and in short FOS. As mentioned, the *B. breve* UCC2003 genome contains a FOS operon, which encodes a putative permease, a conserved hypothetical protein and a β-fructofuranosidase. Transcriptional analysis of this operon in *B. breve* UCC2003 grown in the presence of different carbohydrate sources showed its involvement in the hydrolysis of short-chain FOS [92]. Another class of enzymes involved in the utilization of prebiotics by bifidobacteria includes β-galactosidases. These enzymes are essential for bifidobacteria to be able to grow in milk or milk-components like lactose and lactose-derived transgalacto-oligosaccharides (TOS). Arabinofuranosyl-containing oligosaccharides derived from plant cell-wall constituents, such as arabinan and arabinoxylan, can be fermented by bifidobacteria through arabinoxylan- and arabinofurano-hydrolases [93]. However, the rate of degradation of these compounds is rather low and it has been assumed that other bacteria (e.g. *Bacteroides*) are needed for the complete hydrolysis of these polymers [25]. Bifidobacteria are also known to grow rapidly on soymilk substrates containing large amounts of α-galactosyl-oligosaccharides (GOS) [94]. Enzymes such as α-galactosidases are responsible for the hydrolysis of these types of substrates as well as for the utilization of melibiose and galactomannan.

### Respiration in *L. lactis*

Sugar fermentation was long considered to represent the sole means of energy metabolism available to lactic acid bacteria, producing organic acids (mainly lactic acid) as a final product. While this description is still true, some LAB species exhibit a respiratory capacity in the presence of oxygen and exogenous heme, resulting in the production of greatly reduced amounts of lactic acid [39].

Early evidence for the respiratory capability of LAB was largely overlooked [95], and the initial reports on the respiration in *L. lactis* [38] were essentially based on the analysis of the complete genome sequence of *L. lactis* IL1403 [33]. Subsequent research confirmed that this species certainly has the ability to respire in the presence of oxygen [38, 39], provided the growth medium contains heme because this bacterium does not have a functional biosynthetic pathway for this compound [33]. Heme is an iron containing porphyrin, which is an essential co-factor of the cytochrome oxidase system. An important consequence of respiration is a more efficient conversion of the carbon source into biomass, resulting in higher cell yields and increased survival following growth. Under respiratory conditions acetate, acetoin and diacetyl are produced from pyruvate at the expense of lactic acid [38]. Transcriptomic analysis of *L. lactis* MG1363 showed that the pyruvate dehydrogenase complex (encoded by the *pdhABCD* operon) was up-regulated 4-fold under these circumstances. Acetolactate syntase (*als*) and α-acetolactate decarboxylase (*aldC*) genes were also up-regulated, facilitating the synthesis of both diacetyl and acetoin. The most highly up-regulated gene under respiratory conditions was *ygfC*, encoding a putative regulatory protein [97], which increased almost 100-fold. In contrast, the expression of the pyruvate formate lyase (*pfl*) and the alcohol dehydrogenase (*adhE*) was reduced 2.5- and 50-fold, respectively.

These results are of industrial significance and allowed the development of a patented process for the production of LAB starter cultures [96]. The patent was licensed to Chr. Hansen in 1999, and the initial results obtained with the well-characterized laboratory strain *L. lactis* IL1403 were optimized for different industrial *L. lactis* strains and also for *Leuconostoc* species [40]. Industrial *L. lactis* strains strains were also assayed in aeration in the absence of added heme to distinguish simply aeration from true respiration [40]. Numerous genes were differentially expressed under these two different conditions. Approximately, half of these genes have unknown function, indicating that more research is needed to fully understand the physiology of respiration in this species. However, increased biomass after aerated incubation in the presence of heme was not obtained for *S. thermophilus, L. bulgaricus* and *L. helveticus*. Analysis of the complete genome sequences of these species [18, 19, 20] neither revealed the presence of genes for cytochrome oxidase nor for the biosynthesis of quinones, features which are believed to be essential for respiration [39].

In any case, starter cultures obtained by the respiration technology have been assayed in pilot scale tests for Cheddar cheese production. Manufacture parameters were all within the normal range, and differences between respiration-grown cells and the fermentation-grown cells were not observed [40]. Parameters such as moisture content, total soluble nitrogen or pH did not significantly change after two or six months of ripening. Indeed, sensory differences were not perceived by two trained sensory panels after these periods. Industrial scale trials of Cheddar, Feta and Cottage cheese have already been performed, and again no significant differences were seen in the manufacturing parameters, cheese microbiology, chemistry, texture or flavor development [as reported in 40].

## GENOME DIVERSITY AND STRAIN COMPARISON

Whole genome sequencing, genome data mining, and comparative genomics provide insights into genetic content, differences and similarities among species and strains, and offer important clues into possible gene functions [98]. A powerful method to quickly determine the genome content of a bacterial strain whose genome sequence is not known is comparative genome hybridization (CGH). By this technique, chromosomal DNA fragments of test and sequenced strains, carrying differential fluorescent labels, are hybridized against a DNA microarray that represents all identified genes of the latter strain. CGH is commonly used to determine the genomic composition and genome plasticity of bacteria [99]. The technique is also considered as an alternative to the complete sequencing of genomes, especially in close related species or in strains of the same species [23, 100], because, as the microarray analysis is based on DNA-DNA hybridization, distantly related species lacking enough DNA sequence identity can not be evaluated by this technique. Two different types of genomic diversity are expected, the selfish mobile DNA (referred to as the mobilome), which enters and leaves the genome, and remnants of ancestral DNA, that has not been lost during genome reduction [101].

At present, only a few studies describe the use of CGH to determine the genome composition in LAB species [80, 100]. In one of these studies, DNA microarrays of the *L. lactis* IL1403 were hybridized with a mixture of randomly Cy3- or Cy5-labeled DNA fragments from *L. lactis* IL1403 and *L. lactis* MG1363 [80]. As the genomes of these two strains are available, the competitive hybridization behaviour of the whole gene complements could give some clues about the differences between similarity levels, copy number and hybridization efficiency. A clear positive correlation between gene similarity and the hybridization signal was obtained for genes with a similarity of 75% or higher. However, below this critical level of similarity a relative large scatter in ratios between different genes was observed [80]. Microarrays have also been used to explore the genome diversity of *L. plantarum* strains [100]. DNA microarrays based on *L. plantarum* WCFS1 were hybridized against total DNA from 20 strains. Genes that were present in the WCFS1 strains but not in others were analyzed with respect to their chromosome location, base composition, and putative functions. A high degree of gene content variability was observed among the *L. plantarum* strains examined. The majority of genes found in the variable regions were seen to be involved in sugar metabolism, and based on their unusual base composition and overrepresentation these genes were thought to constitute lifestyle adaptation regions in this bacterium [57, 100].

Similarly, the technique has been used for a comparison of several members of the *L. acidophilus* group against microarrays based on the *L. johnsonii* NCC 533 genome, addressing both intra- and interspecies diversity [101]. A clear stepwise decrease in similarity between members of the *L. acidophilus* complex was found, suggesting the species belong to a natural phylogenetic group. Thus, this technology may also be exploited to clarify the taxonomical relationships in problematic bacterial groups. The intraspecies differences were analyzed by comparing four different strains with the NCC 533 genome. Overall, DNA from the test strains failed to hybridize with 8% to 17% of the ORFs from the reference *L. johnsonii* strain [101]. In contrast to *L. plantarum*, the region around the origin of replication proved to be the largest genome segment of gene conservation. In *L. johnsonii* the region around the replication terminus was shown to be a major area of genetic diversity. Moreover, DNA microarray based on the NCC533 genome revealed that a large proportion of the NCC 533 strain-specific DNA sequences is represented by phage DNA [102].

Genome-wide comparison of nine different *B. longum* strains has recently been reported by this technique [23]. Seven major regions of non-conserved sequences were identified, of which four have a low GC content and had previously been identified by comparison of the genome sequence of NCC 2705 and DJO10A.

## GLOBAL ANALYSIS OF LAB AND BIFIDOBACTERIAL PROTEINS, PROTEOMICS

The proteome is the total protein complement of a genome under specific conditions. In most cases, it is investigated by combining 2D gel electrophoresis and protein identification by peptide mass spectrometry and peptide sequencing using an MS-MS approach [103]. Only in combination with the genome sequence can the proteomics potential be fully exploited, although the peptides obtained can also be directly compared to the ever-increasing protein databases. Like transcriptomic analyses, proteomic studies can generate quantitative measures, of protein levels in this case. Combining transcriptomic and proteomic data it is possible to determine whether regulation takes place at the transcriptional or post-transcriptional level.

Reference maps can also be produced for a mixed bacterial population. For example, Gagnaire *et al*. [104] identified the proteins released into Emmental cheese by strains of *L. helveticus, L. delbrueckii* subsp. *lactis* and *S. thermophilus*. The analysis showed that some peptidases from *L. helveticus* and *S. thermophilus* are released into the cheese matrix, indicating these may contribute to peptide degradation during cheese ripening.

The technique may be very helpful in uncovering complex interactions and mutualistic relationships between LAB/bifidobacterial species and their environment, including the interactive effects between LAB/bifidobacterial and animal or human intestinal cells, which may ultimately lead to a more rational design of probiotics.

## NATURAL TRANSFORMATION OF CERTAIN LAB

Progress in the molecular characterization of important LAB properties to be used as starters or probiotics, and their further improvement by genetic engineering has been traditionally hampered by a lack of genetic tools [105]. Indeed, the genetics of these microbes is poorly developed relative to other microorganisms of industrial significance. Some plasmids were soon recognized as conjugative [82, 106], a capability that was utilized to construct robust industrial starter strains equipped with many phage resistance systems, bacteriocin production, etc. [9, 105]. Transduction was discovered in *L. lactis* even before conjugation [107]. In contrast, natural transformation has never been observed for any of the typical LAB species. (However, as judging for the present shape of the genomes, these two processes might have had a significant role in determination of the gene content of these species). Thus, the introduction of DNA into LAB species initially relied on the formation and regeneration of protoplasts, and subsequently on electrotransformation methods. These were developed for other bacterial types, and then adapted to LAB species [105]. Homologous and heterologous plasmids were developed into plasmid vectors, opening the way to an array of important findings in plasmid- and chromosomally-encoded properties, such as the metabolic abilities that allow these bacteria to utilize lactose and casein, phage resistance, bacteriocin production and immunity, and exopolysaccharide production [9].

Bacteria that are competent for natural genetic transformation are able to take up naked DNA from the environment, and incorporating it into their genomes by (homologous) recombination. Competence is a transient, tightly regulated process, involving two sets of genes: the early and late competence genes. One of the best-studied naturally competent bacteria is *S. pneumoniae *[74]. Early competence genes in this bacterium comprise a quorum-sensing system consisting of ComABCDE, in which *comC* encodes the precursor of a secreted peptide pheromone, the competence-stimulating peptide (CSP), which triggers development of the competent state when its external concentration in the culture reaches a critical threshold. The CSP is secreted by the ABC-type transporter ComAB, acting through a two-component signal transduction pathway consisting of the histidine kinase ComD and the cognate response regulator ComE. Fourteen proteins are known in pneumococci to be necessary for uptake of extracellular DNA and its subsequent incorporation into the recipient’s genome. These 14 proteins are all encoded by late genes. Late genes share an 8-bp sequence in their promoter regions, which are recognized by an alternative sigma factor (ComX). Circumstantial evidence indicates that ComX is encoded by one of the early genes and therefore depends on ComE for expression [108].

Interestingly, recent genome sequencing has shown that the ComX regulon appears to be present in different LAB species, including *L. lactis* [33], *L. plantarum* [57], and all *S. thermophilus* species [18]. This finding suggests that many LAB might actually be naturally transformable provided that growth conditions promoting the development of competence can be identified. Alternatively, these genes may have other functions or represent nonfunctional relics inherited from a shared competent ancestor.

In an elegant study, Blomqvist *et al*. [75] analyzed the expression of the different components of the late competence genes in *S. thermophilus* LMG 18311, which under normal laboratory conditions this strain is not transformable [33]. Blomqvist *et al*. [75] demonstrated that the level of expression of ComX was too low to turn on the competence state. As it had been reported before in *S. pneumoniae *[108], transient overexpression of ComX by an inducible promoter was sufficient to attain transformation efficiencies, using total genomic DNA from a streptomycin-resistant strain, higher than 10^-3^ cfu per donor strain [75]. The mechanism for controlling the development of competence could have degenerated during adaptation of *S. thermophilus* to the dairy niche, but it could also be likely that spontaneous development of competence in this species may require special and as-yet-undiscovered growth conditions. Indeed, evidence of HGT from other dairy bacteria to *S. thermophilus* LMG 18311 has been reported [18]. Thus, it seems plausible that at least some of these gene transfer events have taken place by natural genetic transformation.

In a similar way, a (dormant) natural transformation capacity may be embedded in those LAB species that possess the complete set of competence genes. In light of the promising results of Blomqvist *et al*. [75], natural transformation holds great promise as a novel tool allowing easy, rapid, and efficient construction of food-grade mutants of industrial LAB strains, which may serve for designing starter and probiotic strains with improved properties by attractive, non-controversial techniques for both consumers and dairy industry.

## CONCLUSIONS

The first Lactic-acid producing bacterial species for which the complete genome sequence was publicly available was *L. lactis* subsp. *lactis* IL1404 in 2001 [33]. The genomic information gathered in the last seven years may be useful to gain insights into LAB and bifidobacterial physiology, biochemical potential, as well as their evolution, unraveling how the different species evolved and adapted to their respective ecological niche. This information may be of direct industrial and/or biotechnological application, as it was the case for the respiration process in *L. lactis*. The availability of the genome sequences of many LAB and bifidobacteria will allow global research approaches, such as comparative genomics, transcriptomics, metagenomics, proteomics, and metabolomics, some of which were not covered in this review. All these technologies can be exploited to address a medley of fundamental and applied questions, such as the genetic basis and control of the nitrogen and carbon metabolism, factors involved in host colonization and competition, resistance to antimicrobials and stress responses, crucial traits for the selection of technologically and functionally robust commercial strains. The sequence data provided by the finalized and ongoing genome sequencing projects and the development of more robust high-throughput techniques of DNA and protein analysis will further allow deeper genomic and post-genomic studies, translating sequences into biologically-relevant information.

## Figures and Tables

**Table 1.** General Genome Features of Typical LAB Genomes

Species-Strains	Genome Length, bp	GC Content (%)	Plasmids (No. of Genes)	No. of Proteins	No. of Pseudogenes	No. of rRNA Operons	No. of tRNAs	No. of Prophages	IS Elements (Copies)	[Reference]/ GenBank no.
**Bifidobacteria**										
*B. adolescentis *ATCC 15703	2,089,645	59	0	1,631	None	5	54	?	?	NC_008618
*B. adolescentis *L2-32	2,385,710	59	0	2,428	None	5	54	?	?	AAXD00000000
*B. longum *biovar longum NCC2705	2,256,646	60	pBLO1 (2)^a^	1,727	None	4	57	1?	6 (16)^b^	[22]/NC_004307
*B. longum *biovar longum DJO10A	2,375,286	59	pDOJH10L (10), pDOJH10S (3)	1,908	None	4	57	?	?	AABM00000000
**Lactobacilli**										
*L. acidophilus *NCFM	1,993,564	34	0^c^	1,864	None	4	61	2 remnants	7 (17)	[17]/NC_006814
*L. brevis *ATTC 367	2,340,228	46	pLVIS1 (12), pLVIS2 (25)	2,221	50	5	65	1	(31)	[15]/NC_008497
L. casei *ATTC 334*	2,924,325	46	pLSEI1 (20)	2,776	82	5	59	2	(90)	[15]/NC_008526
*L. delbrueckii* subsp. *bulgaricus* ATCC BAA365	1,856,951	49	0	1,725	192	9	98	0	(33)	[15]/NC_008529
*L. delbrueckki *subsp. *bulgaricus *ATCC11842	1,864,998^d^	49	0	1,562	270	9	95	0	10 (?)	[19]/NC_008054
*L. gasseri *ATTC 33323	1,894,360	35	0	1,763	43	6	78	1	(3)	[15]/NC_008530
*L. helveticus* DPC 4571	2,080,931	38	0	1,848	155	4	73	1 renmant	21 (213)	[20]/NC_010080
*L. johnsonii *NCC 533	1,992,676	34	0	1,821	None	6	79	2	(14)	[56]/NC_005362
*L. plantarum *WCFS1	3,308,274	44	pWCFS101 (3), pWCFS102 (4), pWCFS103 (43)	3,052	39	5	62	2	2 (12)	[57]/NC_004567
*L. reuteri *F275	1,999,618	38	0	2,027	39	6	68	?	?	NC_009513
*L. reuteri *100-23	2,174,299	38	0	2037	2	6	65	?	?	AAPZ00000000
*L. sakei *23K	1,884,661	41	0	1,883	30	7	63	1 remnant	4 (12)	[58]/NC_007576
*L. salivarius* subsp. *salivarius* UCC118	2,133,977	32	pSF118-20 (27), pSF118-44 (52), pMP118 (220)	1,834	73	7	78	4 (2 remnant)	16 (43)	[44]/NC_007929
**Lactococci**										
*L. lactis *subsp. *cremoris* MG 1363	2,529,478	35	Plasmid cured	2,436	81	6	62	6 (4 remnant)	12 (71)	[34]/NC_009004
*L. lactis* subsp. *cremoris *SK11	2,641,635	35	pLACR1 (11), pLACR2 (8), pLACR3 (66), pLACR4 (39), pLACR5 (8)	2,509	153	6	62	4?	9 (130)	[15]/NC_008527
*L. lactis *subsp. *lactis* biovar diacetylactis IL1403	2,365,589	35	Plasmid cured	2,310	1	6	62	6	6 (43)	[33]/NC_002662
**Leuconostoc**										
*L. citreum* KM20	1,796,284	39	pLCK1 (49), pLCK2 (36), pLCK3 (29), pLCK4 (13)	1,820	-	4	69	remnants	2 (10)	[68]/NC_010471
*L. mesenteroides *subsp. * mesenteroides* ATTC8293	2,075,763	37	pLEUM1 (34)	2,009	19	4	71	1	(10)	[15]/NC_008531
**Oenococci**										
*O. oeni* PSU-1	1,780,517	37	0	1,691	122	2	43	0	(?)	[15]/NC_008528
*O. oeni* ATCC BAA-1163	1,753,447	37	0	1,398	277	3	43	0	(9)	AAUV00000000
**Pediococci**										
*P. pentosaceus *ATTC 25745	1,832,387	37	0	1,757	19	5	55	2?	(4)	[15]/ NC_008525
**Streptococci**										
*S. thermophilus* LMD9	1,864,178	39	pSTER1 (2) pSTER2 (4)	1,710	206	6	67	1?	(63)	[15]/NC_008532
*S. thermophilus *CNRZ1066	1,796,226	39	0	1,915	182	6	67	1	(55)	[18]/NC_006449
*S. thermophilus *LMG13811	1,796,846	39	0	1,890	180	6	67	0	(54)	[18]/NC_006448

a Also integrated plasmid remnants.

b Three unique and identical IS structures (three successive integrases inserted in the middle of an IS).

c Three potentially autonomous integrated regions (7x3 ORFs).

d An unusual inverted repeat at the chromosome replication terminus of 47.5 kb.

? , data not reported or not contrasted.
